# BK polyomavirus nephropathy in two kidney transplant patients with distinct diagnostic strategies for BK virus and similar clinical outcomes: two case reports

**DOI:** 10.1186/s13256-017-1300-9

**Published:** 2017-05-24

**Authors:** Ana Luisa Figueira Gouvêa, Rachel Ingrid Juliboni Cosendey, Ana Lucia Rosa Nascimento, Fabiana Rabe Carvalho, Andrea Alice Silva, Heleno Pinto de Moraes, Mayra Carrijo Rochael, Rafael Brandão Varella, Stephanie Gomes Almeida, Jorge Reis Almeida, Jocemir Ronaldo Lugon

**Affiliations:** 1 Laboratório Multiusuário de Apoio à Pesquisa em Nefrologia e Ciências Médicas (LAMAP), Department of Clinical Medicine, Universidade Federal Fluminense, Rua Marquês do Paraná, 303, Niterói, 24033-900 Rio de Janeiro Brazil; 2 Department of Pathology, Universidade Federal Fluminense, Niterói, Brazil; 3 Department of Pathology, Universidade do Estado do Rio de Janeiro, Rio de Janeiro, Brazil; 4 Department of Preventive Medicine, Universidade Federal do Rio de Janeiro, Rio de Janeiro, Brazil

**Keywords:** BK polyomavirus, BK-associated nephropathy, Decoy cell, Urinary monitoring, Renal transplant, Allograft dysfunction infectious disease

## Abstract

**Background:**

BK polyomavirus-associated nephropathy is an important cause of post-transplantation renal failure. We present two cases of BK polyomavirus-associated nephropathy who were submitted to contrasting strategies of clinical follow-up to BK polyomavirus reactivation, but progressed to a similar final outcome.

**Case presentation:**

Case 1 is a 37-year-old white man whose graft had never presented a good glomerular filtration rate function, with episodes of tacrolimus nephrotoxicity, and no urinary monitoring for BK polyomavirus; stage B BK polyomavirus-associated nephropathy was diagnosed by biopsy at 14 months post-transplant. Despite clinical treatment (dosage decrease and immunosuppressive drug change), he progressed to stage C BK polyomavirus-associated nephropathy and loss of graft function 30 months post-transplant. Case 2 is a 49-year-old mulatto man in his second renal transplantation who was submitted to cytological urinary monitoring for BK polyomavirus; he presented early, persistent, and massive urinary decoy cell shedding and concomitant tacrolimus nephrotoxicity. Even with decreasing immunosuppression, he developed BK polyomavirus-associated nephropathy 1-year post-transplant. Loss of graft function occurred 15 months post-transplant.

**Conclusions:**

Cytological urinary monitoring was an efficient strategy for monitoring BK virus reactivation. Decoy cell shedding may be related to BK polyomavirus-associated nephropathy when extensive and persistent. The presence of associated tacrolimus nephrotoxicity may be a confounding factor for the clinical diagnosis of BK polyomavirus-associated nephropathy.

## Background

BK polyomavirus-associated nephropathy (BKVAN) affects up to 15% of renal transplant recipients and is an important cause of graft failure, due to insidious inflammatory destruction of the renal tissue [1–5]. Monitoring of BK polyomavirus (BKV) infection in these patients is required for early detection of reactivation. It can be performed by detection of decoy cells (DC) in urine or detection of virus in plasma and urine using polymerase chain reaction (PCR) tests [5–10]. BKVAN diagnosis depends on specific morphological findings in allograft biopsy or detection of small and cohesive aggregates of polyomavirus called Haufen-polyomavirus in ultrastructural urine tests [11–13].

Many risk factors are involved in BKV reactivation [13–15], but the identification of patients who have high risk to develop BKVAN remains a challenge [16]. Specific antiviral treatments for BKV are not available [14, 17]. The absence of a standard protocol for BKV infection treatment makes clinical management of these patients difficult.

We present two cases of BKVAN. Patient 1 was not monitored for BKV reactivation, while patient 2 was regularly monitored for BKV by urine testing for the presence of DC, which led to early diagnosis of viral reactivation. Both patients lost graft function due to BKVAN approximately a year after transplantation.

## Case presentation

### Case 1

**Fig. 1:**
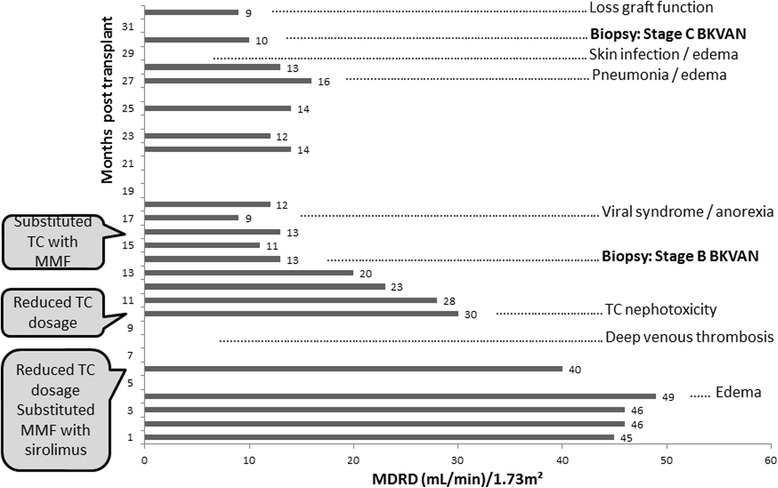
Patient 1. Estimated glomerular filtration rate over 32 month follow-up. The timeline of clinical events and management is indicated. *BKVAN* BK polyomavirus-associated nephropathy, *MDRD* Modification of Diet in Renal Disease Study equation, *MMF* mycophenolate mofetil, *TC* tacrolimus

**Fig. 2:**
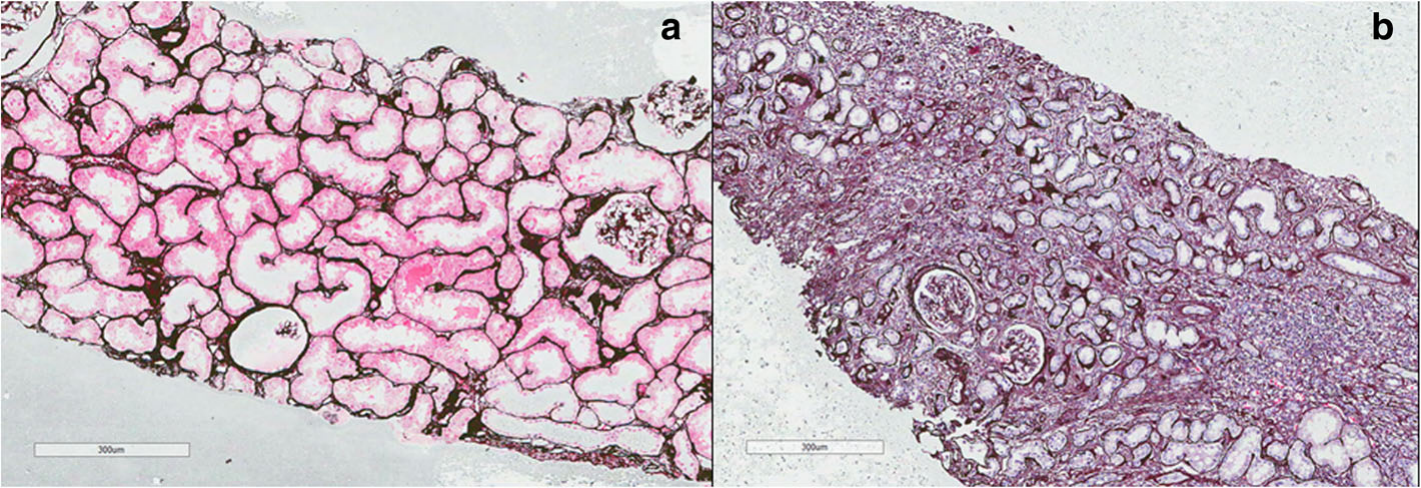
Patient 1. Kidney allograft biopsy: **a** Stage B BK polyomavirus-associated nephropathy; **b** Stage C BK polyomavirus-associated nephropathy. Methenamine silver stain

A 37-year-old white man underwent kidney transplant in July 2013 due to hypertension nephropathy. In the first postoperative (PO) 5 months, his glomerular filtration rate estimated by the Modification of Diet in Renal Disease Study equation (MDRD) [18] ranged between 45 and 49 mL/minute per 1.73 m^2^ (Fig. 1). In PO month 6, he was administered prednisone 5 mg/day, mycophenolate mofetil (MMF) 1440 mg/day, and tacrolimus 6 mg twice a day. The tacrolimus was reduced from 12 mg to 6 mg/day and MMF was substituted with sirolimus (3 mg/day). In PO month 10, there were clinical signs of tacrolimus nephrotoxicity and the dosage was further decreased to 4 mg/day. A graft biopsy was performed in PO month 14, due to continuous decreasing renal function. The biopsy revealed stage B BKVAN characterized by: epithelial tubular necrosis foci; many epithelial cells with basophilic intranuclear inclusions presenting diffusely in the core, especially in the medulla; patchy and moderate interstitial infiltrate with predominance of lymphocytes; a microabscess of neutrophils involving medullar tubules; edema, especially in the medulla; patchy stromal bleeding; few foci of mild tubulitis (maximum two inflammatory cells per tubular cross-section); tubular atrophy; interstitial fibrosis in <10% of renal cortex (Fig. 2a); and arteriolosclerosis with some hyalinosis. Many tubular epithelial cells were positive for Simian virus 40 large T antigen (SV40 T-ag). There were no signs of transplant glomerulopathy or vasculopathy/vasculitis. Tacrolimus was substituted with MMF 720 mg/day. Our patient presented episodes of bacterial infection in his respiratory tract (PO month 27) and skin (PO month 29), when two sessions of hemodialysis were required. A second graft biopsy was performed in PO month 30, showing extensive interstitial fibrosis (>90% renal cortex) and inflammatory cell infiltration (>50% renal cortex), mainly composed of lymphocytes (Fig. 2b). Some tubular cells in the medulla showed basophilic intranuclear inclusions. There were no signs of transplant glomerulopathy or vasculopathy and complement component 4d (C4d) was negative. There was weak nuclear positivity for SV40 T-ag in a few tubular epithelial cells in the cortex and medulla. The diagnosis was stage C BKVAN. He was returned to the hemodialysis program.

### Case 2

**Fig. 3:**
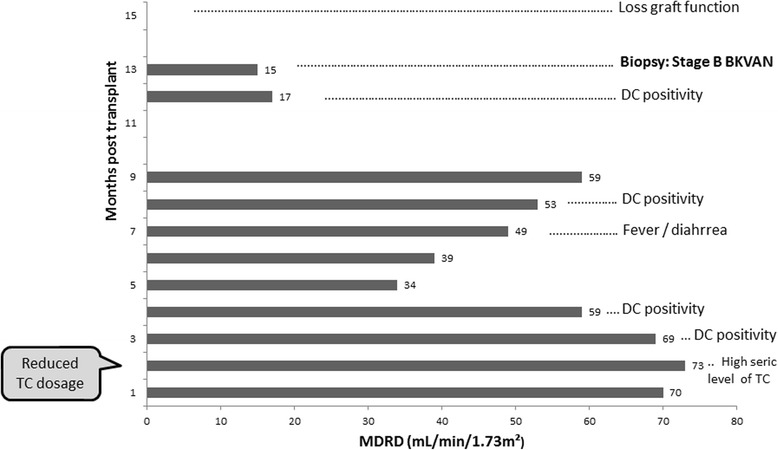
Patient 2. Estimated glomerular filtration rate over 15 month follow-up. The timeline of clinical events and management is indicated. *BKVAN* BK polyomavirus-associated nephropathy, *DC* decoy cells, *MDRD* Modification of Diet in Renal Disease Study equation, *TC* tacrolimus

**Fig. 4:**
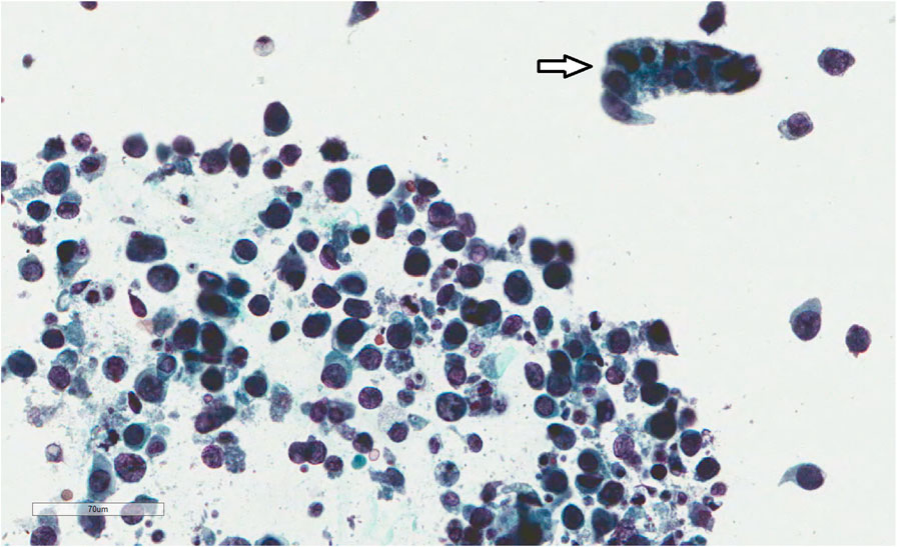
Patient 2. Numerous decoy cells and a decoy cell cast (*arrow*). Papanicolaou stain

**Fig. 5:**
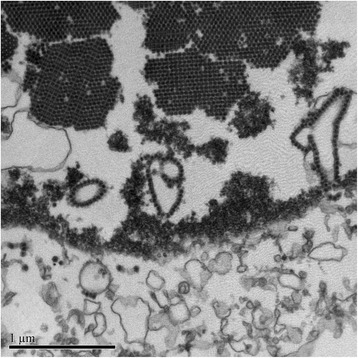
Patient 2. Decoy cell ultrastructure: chromatin clumps at nuclear periphery and intranuclear spherical virions arranged in crystalloid array

**Fig. 6:**
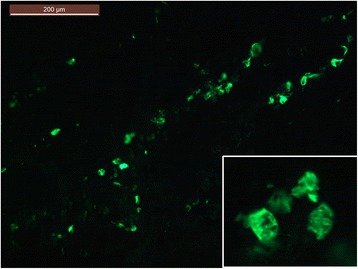
Patient 2. Kidney allograft biopsy: Simian virus 40 large T antigen nuclear staining in renal tubular cells. *Inset*: nuclear detail (immunofluorescence)

A 49-year-old mulatto man underwent kidney transplant in June 2015 due to autosomal dominant polycystic kidney disease. He underwent a previous kidney transplant in 2011 and spent 3 years on dialysis between the two transplants. In PO month 2, his serum tacrolimus level was 26.7 ng/mL. He was administered prednisone (15 mg/day), sirolimus (3 mg/day), and tacrolimus (2 mg twice a day). Tacrolimus was decreased from 4 mg to 2 mg/day (Fig. 3). He was submitted to biweekly urinary monitoring for BKV (screening for DC in urine sediment). Urinary DC were detected in PO month 3. From then on, all urine samples were strongly positive for DC, >10/high power field (HPF), with the smears presenting a dirty background, cellular debris, many leukocytes and, in some samples, cellular casts with nuclei showing features of polyomavirus infection (Fig. 4). An ultrastructural study of urine sediment revealed abundant icosahedral viral particles measuring approximately 40 nm in diameter, either intranuclear or in the cytosol, as single particles, small aggregates, or forming typical crystalline arrays, free or membrane-bound, or extracellular in continuity with the cell membrane (Fig. 5). In PO month 12, there was a sharp drop in renal function and a graft biopsy was performed. The histology revealed stage B BKVAN characterized by tubular cellular necrosis associated with many nuclear inclusions expressing SV40 T-ag (Fig. 6), present in both cortex and medulla, minimal interstitial inflammatory infiltrate, fibrosis and tubular atrophy, no tubulitis, and no transplant glomerulopathy or vasculopathy. There were no typical morphological signs of nephrotoxicity by tacrolimus. He returned to the hemodialysis program in PO month 15.

## Discussion

Effective and safe antiviral therapies for BKVAN are not available. The management of patients with BKV reactivation is a challenge because there is not a reliable and universally accepted protocol to follow.

The pathogenesis of BKVAN is multifactorial, with several known risk factors: immunosuppression (a general prerequisite); “high dose” of new drugs such as tacrolimus; pre-transplant use of antilymphocyte therapy and MMF use at baseline; tubular injury/regeneration and/or ischemia/reperfusion in allograft (since native kidneys are generally not involved); human leukocyte antigen (HLA) mismatches; recipient diabetes; previous acute rejection; recipient age >55 years; recipient race (white); and recipient gender (male) [4, 5, 14, 15, 19–21]. Both cases discussed here are male, presenting tacrolimus nephrotoxicity before BKVAN development. Higher blood levels of tacrolimus are associated with an increased incidence of BK viremia, which supports the notion that immunosuppression increases the risk of BKVAN [14]. Epithelial cell proliferative state in response to different forms of injury may increase BKV replication [15]. When their charts were reviewed, both patients presented previous tubular damage (multifocal epithelial necrosis) in their pre-implantation donor kidney biopsies.

Once BKVAN is diagnosed, definition of the presence of concurrent rejection can be very difficult to establish, because some morphological aspects may be shared by both conditions and they may co-occur [15, 22]. Some clues for BKVAN diagnosis are: a heterogeneous inflammatory reaction, sometimes minimal, present especially in the medulla and composed of mononuclear cells; polymorphonuclear leukocytes, which can be seen in response to urinary leakage from damaged tubules; and inconspicuous tubulitis and viral lesions restricted to the medulla [19]. On the other hand, in concurrent rejection there is abundant tubulitis, cortical inflammatory infiltrates (more pronounced in areas without viral inclusions), transplant endarteritis, glomerulitis, glomerulopathy, sclerosing vasculopathy, and C4d deposition along the peritubular capillaries [19, 23]. Patient 1 exhibited interstitial edema and hemorrhagic foci. Although no morphological criteria for rejection (Banff classification) were present in this sample, it would be very difficult to definitely rule out this association.

It is difficult to make predictions about the progression of BKVAN. The risk of graft loss function in stage A is <10%, in stage B close to 50%, and in stage C >80% [5]. Both patients were diagnosed at stage B.

Tacrolimus is a calcineurin inhibitor and some histological lesions have been associated with its chronic use, such as striped interstitial fibrosis, tubular atrophy, medial arteriolar hyalinosis, and tubular microcalcification [24]. Tacrolimus may have played a role in the fibrosis observed in the graft biopsies of both patients.

Patient 2 presented early DC shedding. Considering that he had two renal transplants in 4 years, we wondered if BKV reactivation could be linked to his first kidney transplant. A previous transplant, in general, has not been confirmed as a risk factor for BKVAN [21]. However, retransplantation after a graft loss due to BKVAN may be an important factor [5]. Unfortunately, the reason for the loss of his first graft is unknown.

PCR tests for BKV in urine and plasma have higher positive predictive value for BKVAN than urinary cytology. However, urinary cytology is a low cost and simple method for BKV screening, with negative predictive value for BKVAN of 100% [5, 25]. Some authors value the number of DC (>10/HPF), presence of a necro-inflammatory background in urine containing DC, persistent DC shedding (over 6 weeks), and detection of DC casts to identify patients with possible active BKVAN [1, 26, 27]. Patient 2 presented all of these characteristics, but we only had an unequivocal criterion to perform graft biopsy when a clear drop in renal function was detected. The choice of the ideal moment to perform a renal allograft biopsy in BKV infection is a matter of controversy. It is an invasive procedure, which requires strict indication. On the other hand, initial BKVAN can have an indolent presentation, with no clear allograft dysfunction [5]. The associated tacrolimus nephrotoxicity may have masked the real importance of BKV reactivation in the clinical scenario of patient 2.

Both patients were diagnosed with BKVAN at the end of the first year post-transplant. The diagnosis of viral reactivation was conceivably earlier in patient 2, who was systematically monitored. In spite of that, the course of BKVAN in the two cases was similar. An early diagnosis can be very important to preserve the tissue from inflammation and fibrosis. It is unclear if the availability of an effective antiviral drug would have made a difference in the clinical course of these patients.

## Conclusions

Urinary monitoring for DC is a simple and efficient strategy for routine screening of BKV reactivation. Early detection of BKV infection in patients who have undergone a renal transplant is crucial to identify patients demanding closer clinical supervision. The presence of massive and persistent DC shedding can indicate a high risk for BKVAN development, even if renal function is normal.

Tacrolimus nephrotoxicity is a common complication in patients who have undergone a renal transplant and may mask the real importance of BKV reactivation.

## Abbreviations

BKV: BK polyomavirus
BKVAN: BK polyomavirus-associated nephropathy
C4d: Complement component 4d
DC: Decoy cells
HLA: Human leukocyte antigen
HPF: High power field
MDRD: Modification of Diet in Renal Disease Study equation
MMF: Mycophenolate mofetil
PCR: Polymerase chain reaction
PO: Postoperative
SV40 T-ag: Simian virus 40 large T antigen
